# 

**Published:** 1892-04

**Authors:** 


### CONTENTS.

ORIGINAL ARTICLES—Morbid Reflex Neurosis—By Willis F. Westmoreland, M. D., Atlanta, Ga. .......169
Clinical Lecture—By G. Frank Lydston, Chicago. .......178
The Treatment of Gall Stone, with Cases—By W. E. B. Davis, M. D., Rome, Ga. .......182
Malaria Hæmoglobinuria—By H. E. Williams, M. D., Fordyce, Ark. .......184
SOCIETY NOTES—Atlanta Society of Medicine. .......188
Clinical Society of Maryland. .......191
BOOK REVIEWS. .......197–199
A Practical Treatise on Diseases of Women—By T. Gaillard Thomas, M. D., and Paul F. Munde, M. D.—Quiz Compends on Physiology—By Dr. Brubrake—Annals of the Universal Sciences—Edited by Charles E. Sojous, M. D.,—Saunders' Pocket Medical Formulary—International Clinics.
SELECTIONS AND ABSTRACTS. .......200–210
Vulvitis and Vulvo-Vaginitis in Young Girls—How to Administer Iron Solution of Bromide of Strontium—Reflex Irritations and Neuroses Caused by Stricture of the Urethra in the Female—Pure Strontium Salts—The Responsibility of Sexual Pervert—Poisoning by Cocaine—Leucorrhœa—International Periodical Congress of Gynæcology and Obstetrics—New Buildings for the Jefferson Medical College of Philadelphia—Notes Relative to the Buffalo Lithia Water.
EDITORIALS. .......211–216
Railroad Surgeon at Cut Rates for Service—Railroad Surgeons—Preliminary Programme of the Medical Association of Georgia.
PRESCRIPTION DEPARTMENT. .......219–220
Nervous Dyspepsia—Convulsions of Teething—Swollen Eyes—Taxative for Children—To Abort Boils—For Cough in Neuralgia Cephalalgia—Incontinence of Urine—Pruritis.
SPECIAL NOTES. .......217–219
Second Hand Books—Ingluvin—Sanders & Sons—Cactina Pillets—Jerome Kidder & Co—Sharp & Dohme—Renz & Henry—E. Fougera & Co.

